# Bivariate variance-component analysis, with application to systolic blood pressure and total cholesterol levels in the Framingham Heart Study

**DOI:** 10.1186/1471-2156-4-S1-S81

**Published:** 2003-12-31

**Authors:** Jisheng S Cui, Leslie J Sheffield

**Affiliations:** 1 Centre for Genetic Epidemiology, School of Population Health, The University of Melbourne, Parkville, Australia; 2 Murdoch Childrens Research Institute, Royal Children's Hospital, The University of Melbourne, Parkville, Australia

## Abstract

**Background:**

The correlations between systolic blood pressure (SBP) and total cholesterol levels (CHOL) might result from genetic or environmental factors that determine variation in the phenotypes and are shared by family members. Based on 330 nuclear families in the Framingham Heart Study, we used a multivariate normal model, implemented in the software FISHER, to estimate genetic and shared environmental components of variation and genetic and shared environmental correlation between the phenotypes. The natural logarithm of the phenotypes measured at the last visit in both Cohort 1 and 2 was used in the analyses. The antihypertensive treatment effect was corrected before adjustment of the systolic blood pressure for age, sex, and cohort.

**Results:**

The univariate correlation coefficient was statistically significant for sibling pairs and parent-offspring pairs, but not significant for spouse pairs. In the bivariate analysis, the cross-trait correlation coefficients were not statistically significant for all relative pairs. The shared environmental correlation was statistically significant, but the genetic correlation was not significant.

**Conclusion:**

There is no significant evidence for a close genetic correlation between systolic blood pressure and total cholesterol levels. However, some shared environmental factors may determine the variation of both phenotypes.

## Background

In an early analysis of the Framingham Heart Study data, it was observed that the modest correlation in blood pressure between spouses was reduced when blood pressure was adjusted for weight [1]. This finding suggested that environmental factors shared by spouses might influence blood pressure and related phenotypes. In an analysis based on data from the Victorian Family Heart Study [2], Cui and colleagues used cross-trait correlation and bivariate variance component analysis techniques to investigate the genetic and shared environmental correlations between systolic blood pressure (SBP) and diastolic blood pressure (DBP) and between SBP and body mass index (BMI) [3]. They found that the same genes and many of the same family living environmental factors determine variation in both SBP and DBP. On the other hand, SBP and BMI share genetic and family environmental factors to a lesser degree.

In this article, we applied similar analysis techniques to investigate the genetic and shared environmental components of variance between SBP and total cholesterol levels (CHOL) based on data from the Framingham Heart Study.

## Methods

A total of 330 nuclear families, comprising 1776 individuals (879 males and 897 females), were used in this analysis. When there are multiple nuclear families in a pedigree, a nuclear family that has at least one offspring in Cohort 2 was randomly selected for the analysis. We used the phenotypes of SBP and CHOL, which were measured at the last visit, and transformed them into the logarithms. This ensured that their distributions were close to the normal distribution. We also used the corresponding ages at the last visit in the analysis. A total of 72 parents (31 fathers and 42 mothers) did not have any information on age and the phenotypes. This accounted for 4% (72/1776) of all individuals. These parents did not receive any antihypertensive treatment. There are no missing values in age and the phenotypes in the offspring generation. We analyzed the data with and without imputation. To correct for the antihypertensive treatment effect, we added 10 mm Hg to the measured SBP for the treated subjects and used the original measurements for the untreated subjects [3,4]. We used the software Stata [5] and S.A.G.E. [6] to calculate the descriptive statistics and check the pedigree data.

Statistical analyses were carried out under a multivariate normal model for pedigree analysis using the software FISHER [7,8]. The mean phenotypes were adjusted for age, sex, and their interaction in each cohort. The variances and correlations between relatives were estimated under maximum likelihood theory. Standard errors and 95% confidence intervals were calculated by using the large sample normal approximation [9]. The variance of each phenotype *Y *was given by σ_a_^2 ^+ σ_se_^2 ^+ σ_e_^2^, where σ_a_^2^, σ_se_^2^, and σ_*e*_^2 ^are the genetic, shared environmental, and individual-specific environmental variances, respectively.

### Univariate analysis

Univariate variance component analyses were conducted for each phenotype separately. The covariance between a pair of individuals depends on the type of relationship. For non-spouse relationships the covariance is given by 2φσ_*a*_^2 ^+ γσ_*se*_^2^, where φ is the kinship coefficient between the two individuals and γ (-1 ≤ γ ≤ 1) is the coefficient for shared environmental effect. We estimated the coefficients for sibling pairs, γ_*Sib*_, and for parent-offspring pairs, γ_*PO*_, from the data. The covariance between spouses was modeled separately by ρ_*SP*_(σ_*a*_^2 ^+ σ_*se*_^2 ^+ σ_*e*_^2^), where ρ_*SP *_is the correlation coefficient between spouses for that trait.

### Bivariate analysis

As an extension to the univariate analysis, bivariate variance component analysis considers correlation of two phenotypes simultaneously. The covariance of two phenotypes for an individual was given by ρ_*a*_σ_*a*1_σ_*a*2 _+ ρ_*se*_σ_*se*1_σ_*se*2 _+ ρ_*e*_σ_*e*1_σ_*e*2_, where ρ_*a*_, ρ_*se*_, and P_*e *_are the correlation coefficients for genetic, shared environmental, and individual environmental effect between the phenotypes, respectively. Correspondingly, σ_*ai*_, σ_*sei*_, and σ_*ei *_are the genetic, shared environmental, and individual-specific environmental standard deviations of phenotype *Y*_*i *_(*i *= 1, 2).

The covariance of the two phenotypes between a pair of individuals was parameterized in terms of the different types of relationships. For non-spouse relationship, the covariance was 2φρ_*a*_σ_*a*1_σ_*a*2 _+ γ_1_γ_2_ρ_*se*_σ_*se*1_σ_*se*2_, where γ_*i *_is the coefficient for shared environment of phenotype *Y*_i_(*i *= 1, 2). As for univariate analyses, we estimated γ_*Sib *_and γ_*PO *_separately from the data for sibling and parent-offspring relationships, respectively. For spouse-spouse relationship, the covariance was given by R_SP_σ_1_σ_2_, where σ_*i *_is the total standard deviation of phenotype *Y*_*i *_(*i *= 1, 2) and *R*_*SP *_is the cross-trait correlation coefficient between spouses.

## Results

### Descriptive statistics

Based on data before imputing the missing values, the mean age was 72.5 years (SD 9.3) for the parent cohort and 48.9 years (SD 10.8) for the offspring cohort. Within each cohort, there was no significant difference in age between males and females. Systolic blood pressure (SBP) was significantly higher in the parental cohort, who have a mean of 148 mm Hg (SD23.9), than in the offspring cohort, who have a mean of 124 mm Hg (SD 19.9), based on two-sample t-test (*P *< 0.001). Within each cohort, there was no significant difference in SBP between males and females. Total cholesterol levels (CHOL) were significantly higher (*P *< 0.001) in parents who have a mean of 219 mg/dl (SD 41.0) than in offspring who have a mean of 119 mg/dl (SD 37.2). Among the parents, total cholesterol levels were significantly higher (*P *= 0.001) in mothers who have a mean of 229 mg/dl (SD 40.6) than in fathers who have a mean of 209 mg/dl (SD 39.1). There was no statistically significant difference in CHOL between males and females in the offspring cohort.

### Univariate analyses

Table 1 shows univariate correlation coefficients and their 95% confidence intervals for ln(SBP) and ln(CHOL) in different relative pairs. The magnitude of the correlations was greatest for sibling-sibling pairs in both phenotypes, and least for spouse pairs. These differences between different relative pairs reflect the different impacts of genetic and environmental effects on these phenotypes. Siblings share more living environments than their parents; whereas parents and offsprings are more genetically related than the spouses. The correlations between sibling pairs and between parent-offspring pairs were statistically significant because the confidence intervals did not contain 0. However, the correlation between spouse-spouse pairs was not statistically significant for either phenotype. Table 2 shows the univariate genetic and shared environmental components of variance and 95% confidence intervals for ln(SBP) and ln(CHOL). For ln(SBP), the genetic and shared environmental components of variance accounts for 13% and 11% of the total variance, respectively. The remaining 76% was accounted for by individual-specific factors. For ln(CHOL), a greater proportional of total variance (25%) comes from the shared environmental component, and the genetic and individual-specific components accounted for 5% and 70%, respectively. For shared environmental factors, the coefficients were greater among siblings (γ_*Sib*_) than among parent and offsprings (γ_*PO*_) in both phenotypes. This suggests that individuals within the same generation share more environmental factors than those between generations.

### Bivariate analyses

Table 3 shows the within-individual correlation between ln(SBP) and ln(CHOL), which was 0.24 (95% CI, 0.20–0.29), and the cross-trait correlation for different relative pairs. The cross-trait correlations were not statistically significant for all relative pairs because the confidence intervals contain 0. The correlation coefficient was not significantly higher in parent-offspring pairs than in spouse-spouse pairs. This suggests that there may not be common genetic factors determining both ln(SBP) and ln(CHOL). Table 3 also shows the genetic and shared environmental correlation coefficients between ln(SBP) and ln(CHOL). There seems to be no genetic correlation between these two phenotypes because of the confidence interval containing 0. However, the shared environmental correction was statistically significant, being 0.325 (95% CI, 0.075–0.576). This suggests that some shared family environmental factors determine variation of both phenotypes.

### Imputation of missing values

There are 72 parents with missing values (the two phenotypes and the corresponding age). We imputed these missing values, re-analyzed the data, and compared the results with the above analyses when the missing values were not imputed. The missing value of the parent's phenotypes were generated according to cohort- and sex-specific normal distribution of relevant non-missing phenotypes. The missing age was calculated according to the spouse's non-missing age, which is available for at least one spouse. The husband's age was imputed by adding 2.8 years onto the wife's age, and vice versa. No significant difference in the mean SBP and CHOL in each cohort and gender category was found before and after the imputation. For example, the mean SBP of the parents was 147.3 mm Hg (SD 23.85) after the imputation compared with 147.7 mm Hg (SD 23.87) before the imputation. Correlation and variance component estimates from both univariate and bivariate analyses were very similar to those given in Table 1,2,3. Because only 4% of individuals have missing values, the imputation did not have large impact on the analysis results.

## Discussion

Different statistical analysis methods were used in this paper to investigate the genetic and shared environmental correlations between ln(SBP) and ln(CHOL) based on nuclear families from the Framingham Heart Study. We found significant correlation in sibling-sibling pairs and parent-offspring pairs for ln(SBP) and also for ln(CHOL). No significant correlation was found in spouse-spouse pairs (Table 1). Both univariate and bivariate analyses showed no significant evidence for a close genetic correlation between ln(SBP) and ln(CHOL), but there seems to be some common environmental determinants for both phenotypes. The variation of these two phenotypes may be determined by different genes and by some shared family living environment. This is similar to observations of Cui et al. [3] with regards to SBP and BMI.

However, the two measurements of blood pressure (SBP and DBP) shared both genetic and environmental factors [3]. The Framingham Heart Study involved families that were relatively older than the population studied by Cui et al. [3]. The mean age of that population was 53.8 years (SD 6.3) for the parent and 24.0 years (SD 3.7) for the offspring generation. The sample size of the previous study (767 families) is more than twice the size of this analysis. Another important difference between these two studies is the availability of zygosity data on twins in the study. There is no information about whether some of the siblings are twins in this study. However, the previous study includes 66 monozygotic twins and 84 dizygotic twins. These differences may contribute significantly to the power of this analysis to investigate the genetic and shared environmental correlations and variance components.

Cui et al. [10] have discussed different methods for adjusting hypertensive treatment effects. A fixed value, such as 10 mm Hg, can be added to the measured SBP if a person receives antihypertensive treatment. In contrast, a randomly generated SBP value may be substituted for the measured phenotype for medicated individuals, or the medicated subjects may be totally excluded in variance component and linkage analyses [11,12]. Cui and colleagues found that adding back an appropriate increment of pressure restores familial components and increases the power of genomic linkage analyses to detect quantitative trait loci [10].

## Conclusion

From this investigation, we did not find any significant evidence to suggest that variation in systolic blood pressure and total cholesterol levels is determined by the same genes. However, there seems to be some common family living environment factors determining the variation in both phenotypes. This information is important in considering analysis methods and components of variance for linkage analysis to discover new genes for SBP using the Framingham Heart Study data.

## Figures and Tables

**Table 1:** Univariate correlation coefficients and 95% confidence intervals for ln(SBP) and ln(CHOL) in different relative pairs

		Correlation	
		
Relative pair	N (pairs)	ln(SBP)	ln(CHOL)
Sibling-sibling	1640	0.26 (0.21, 0.30)	0.19 (0.15, 0.24)
Parent-offspring	1964	0.15 (0.11, 0.19)	0.12 (0.08, 0.17)
Spouse-spouse	258	0.09 (-0.04, 0.21)	0.03 (-0.09, 0.15)

**Table 2:** Univariate genetic and shared environmental components of variance confidence intervals for ln(SBP) and ln(CHOL)

Parameter	ln(SBP)	ln(CHOL)
Genetic (1000 × )	2.9 (2.1, 3.7)	1.8 (0.86, 2.7)
Environmental (1000 × )	2.4 (1.3, 3.5)	8.4 (7.0, 9.8)
Individual (1000 × )	16.7 (15.6, 17.8)	23.3 (21.5, 25.1)
Total (1000 × σ^2^)	22.0	33.5
Sibling (γ_*Sib*_)	0.96 (0.87, 1.0)	0.63 (0.56, 0.70)
Parent-offspring (γ_*PO*_)	0.36 (0.22, 0.50)	0.37 (0.30, 0.44)

**Table 3:** Bivariate correlation coefficients and 95% confidence intervals for ln(SBP) and ln(CHOL) indifferent relative pairs

Relative pair	Correlation
Within individuals	0.24 (0.20, 0.29)
Sibling-sibling	0.047 (-0.020, 0.096)
Parent-offspring	0.008 (-0.031, 0.048)
Spouse-spouse	-0.030 (-0.110, 0.050)
Genetic (ρ_*a*_)	0.024 (-0.229, 0.278)
Environmental (ρ_*se*_)	0.33 (0.075, 0.58)
Individual (ρ_*e*_)	0.043 (0.001, 0.086)
